# The Effects of Depression and Fear in Dual-Income Parents on Work-Family Conflict During the COVID-19 Pandemic

**DOI:** 10.1177/21582440231157662

**Published:** 2023-02-27

**Authors:** Gijung Jung, Ji Sun Ha, Mihyeon Seong, Ji Hyeun Song

**Affiliations:** 1 Seoul National University of Hospital, Seoul, Republic of Korea; 2 Busan Institute of Science and Technology, Busan, Republic of Korea; 3 Chang Shin University, Changwon-si, Gyeongsangnam-do, Republic of Korea; 4 Cheju-Halla University, Jeju-si, Jeju-do, Republic of Korea

**Keywords:** depression, fear, family conflict, COVID-19

## Abstract

This study investigated depression and fear in dual-income parents during the COVID-19 pandemic as predictors of work–family conflict. Using a cross-sectional design, we recruited 214 dual-income parents aged 20 years or older with preschool and primary school children in Korea. Data were collected via an online survey. In the final model for hierarchical regression analysis, the strongest predictor of work–family conflict was depression (β = .43, *p* < .001), followed by fear (β = .23, *p* < .001), then weekly working hours (β = .12, *p* < .05). The final model was statistically significant (*F* = 29.80, *p* < .001), with an explanatory power of 35%. These findings highlight the need to provide dual-income parents with government-led disaster psychological support during COVID-19, such as counseling, education, and mental health management services involving the psychological predictors of work–family conflict. Diverse systematic intervention programs and policy support should also be provided to help them resolve work–family conflict.

## Introduction

In response to the global spread of the coronavirus disease 2019 (COVID-19), the World Health Organization declared COVID-19 a pandemic, and as of May 14, 2021, the number of confirmed COVID-19 cases worldwide has reached 160 million (World Health Organization, 2021). South Korea reported 747 new cases and a cumulative total of 130,000 cases as of May 14, 2021 (Statistics Korea, 2021). In response to this long-drawn-out crisis, the Korean government has revived certain social distancing measures, such as banning private gatherings of five people or more, limited business hours for restaurants, and banning social gatherings, as preventive coping strategies against COVID-19 (Korea Disease Control and Prevention Agency, 2021). The pandemic has led to diverse and unprecedented changes taking place in both the society and the economy of the country.

According to international studies, the COVID-19 pandemic has caused many work-related conflicts and restrictions, especially for dual-income parents with preschool (Petts et al., 2021) or school-age children (Heggeness, 2020). Sudden and significant changes to parents’ work and family lives (Shafer et al., 2020) have led to negative workplace outcomes, such as dismissal and high turnover intention (Vaziri et al., 2020). Childcare has also become increasingly burdensome as more children are staying at home during the pandemic (Chin et al., 2020). In Korea, the implementation of social distancing policies on February 27, 2020 led to the repeated closure and re-opening of daycare centers, and since the second half of 2020, local governments have been mandated to determine the closure of daycare centers (Shin et al., 2021). The Korea Ministry of Education announced the fourth postponement of school opening in March 2020, and elementary, middle, and high schools began online classes from April 2020 (Chin et al., 2020). Additionally, stricter social distancing measures were enforced in the Seoul metropolitan areas with a higher incidence of COVID-19 from February to April 2021 (M. Kim, 2021). Since July 2021, level 4 the highest level of the four-tier social distancing system has been enforced in the Seoul metropolitan area, and level 3 has been enforced in all the other regions, resulting in the repeated closure and re-opening of daycare centers and online education for elementary, middle, and high school students. As closure of daycare centers, online classes, and consequent teleworking of parents have hindered families from going outside, families are increasingly experiencing psychological difficulties different from those experienced during the pre-COVID period (B. Lee & Lee, 2021; D. H. Lee et al., 2020). Neologisms, such as *corona blue* (depression, anxiety), *corona red* (anger), and *corona black* (frustration, despair) have emerged to describe the mental health problems induced by COVID-19 (Y. S. Lee, 2020). These can be considered a consequence of working parents’ susceptibility to the care gap in carrying out their responsibilities toward both their work and childcare in situations where children need more parental care. In this context, special attention needs to be paid to help parents maintain work–family balance, and overcome work–family conflict and psychological difficulties encountered during the COVID-19 crisis.

Work–family conflict occurs when role pressures in work and family domains are incompatible for various reasons (Greenhaus & Beutell, 1985). Conflicts in these two domains are interrelated, rather than isolated concepts. From the perspective of the spillover theory, the most representative of the theories explaining work–family conflict, positive or negative emotions or attitudes experienced in these two domains have their respective effects on work and family. In this respect, previous studies have identified the factors affecting work–family conflict in dual-income couples as follows: working hours (Schooreel & Verbruggen, 2016); sleep (Magee et al., 2018); support from friends and colleagues (Wang & Tsai, 2014); gender, age, and number of children; social support, such as family assistants or housekeepers; occupational characteristics (Ayodeji & Akinbode, 2017), parenting stress, depression, marital conflict (Hong & Lee, 2020), and the like. Among the numerous factors surrounding dual-income parents, psychological characteristics, such as anxiety, fear, and depression, can act as risk factors for work–family conflict (Hong & Lee, 2020). For example, a 7-fold increase from 3.44% was reported in the global prevalence of depression in 2017 (Bueno-Notivol et al., 2021). Therefore, it is necessary to improve these psychological characteristics, especially considering the significant impact of the COVID-19 pandemic on mental health (Soraci et al., 2022). Moreover, particular attention needs to be paid to how they affect work–family conflict in dual-income couples.

A significant proportion of previous research, including many studies conducted in Korea, has examined the effect of work–family conflict on emotions. Other studies, however, have reversed this relationship and investigated the effect of emotions on work–family conflict. One study noted that people can encounter work–family conflict caused by work or family stress when they experience negative emotions (Stoeva et al., 2002). Another reported that, on the one hand, low perceived psychological well-being can be a risk factor for work–family conflict, which depression can be a significant determinant of (Hong & Lee, 2020). On the other hand, a study evaluating the longitudinal association between depression and family conflict reported a significant association between depression-related changes and family conflict over time (Oh et al., 2014). In addition to depression, fear is a major factor in an individual’s psychological experience when a novel infectious disease such as COVID-19 emerges (D. H. Lee et al., 2020). Fear is also explained as a variable closely associated with depression (Cori et al., 2021; Sakib et al., 2021). Apart from the fear of COVID-19 as a pandemic, the fears of dual-income mothers have been reported to provoke conflicts between childcare and the workplace (Yang & Shin, 2011). Further, a study found that an increased fear of COVID-19 elevated work–family conflict, although it was not conducted on dual-income parents (Karakose et al., 2021). Moreover, people may develop negative emotions when they cannot control their lives, as with COVID-19, and the fear of infection, in particular, may trigger family conflict (National Center for Mental Health, 2021). In fact, a phenomenological study on mothers of elementary school students in Korea (Moon et al., 2021) reported that the participants had more intense fears about COVID-19 having become a part of their ordinary lives. Moreover, they were afraid of having to continue their roles at home and work which provoked various types of conflicts and caused difficulties in all aspects of their life.

**Figure 1.**
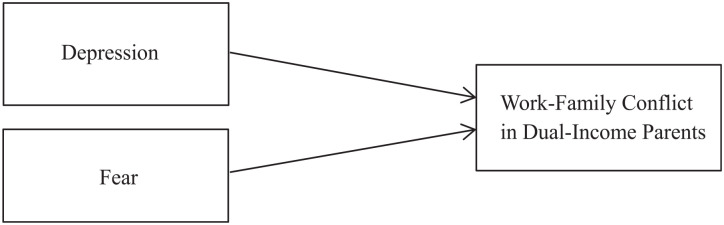
Research framework.

Negative emotions, such as depression and fear, can make individuals more vulnerable to work–family conflict (Allen et al., 2012), and efforts should be made to resolve this problem during the COVID-19 crisis when such psychological factors can have negative effects. This study aims to examine the association between depression, fear, and work–family conflict in dual-income couples with preschool and primary school children during the COVID-19 pandemic. In Korea, a work–life balance culture has not yet been established, and dual-income families continue to experience the difficulties caused by the double burden of work and family lives. Hence, it is important to focus on dual-income parents who have to play multiple roles in a social system, as in Korea. As the generation living with COVID-19, the maintenance of work–life balance in dual-income couples raising children is an urgent task. To this end, this study was conducted to provide basic data to develop intervention strategies for managing negative emotions and resolving work–family conflict. We established the following hypotheses (Figure 1):

Hypothesis 1: Depression is significantly associated with work–family conflict in dual-income parents.
Hypothesis 2: Fear is significantly associated with work–family conflict in dual-income parents.

## Materials and Methods

### Participants

We recruited Korean dual-income parents aged 20 years or older with at least one child who is a sixth grader or younger (S. Kim & Kim, 2013). The recruitment of parents with two or more children was determined by the age of the youngest child (Page et al., 2018). Dual-income parents who were on leave and respondents whose answers were incomplete were excluded from the analysis. The number of subjects was calculated using the G*Power 3.1.9.2 program (Faul et al., 2009), and the number of participants was determined based on the effect size of 15, significance level of .05, and power of .95. The total number of variables (*n* = 14) for general characteristics and study-specific independent variables was 194. We enrolled 214 participants in the study, considering a dropout rate of 10%.

### Procedure

This descriptive survey was conducted after obtaining an exemption from the Institutional Review Board (IRB) of S University in Korea (SM-202102-005-2). Given the difficulty of conducting a face-to-face questionnaire survey with dual-income parents with childcare responsibilities during the pandemic, we commissioned the survey to an online survey agency. The agency developed an online questionnaire based on the IRB-approved questionnaire upon our review, convenience sampled the participants, and began collecting data from April 2021. In accordance with the IRB-approved online data collection protocol, an information sheet explaining the purpose, procedure, duration, confidentiality, and voluntary participation of the study was posted, and only those who voluntarily consented to participate in the study could proceed with the survey. More specifically, the information sheet was shown on the first screen upon accessing the questionnaire link, and the participants had to mark the box to express consent. To protect personal information, no personally identifiable information was collected, and the participants were only given an identification number. We received the collected data as a password-protected Excel file, and changed the password to only allow access to our research team. All research steps were conducted in compliance with the most recent version (revised in 2013) of the Declaration of Helsinki and the ICH-GCP. In addition, all tools used in this study were distributed after obtaining permission from their respective authors.

### Measures

#### The Center for Epidemiological Studies-Depression Scale (CES-D)

Depression perceived by the participating dual-income couples during COVID-19 was measured using the Korean version of the questionnaire (Chon, 1992) adapted from the Center for Epidemiological Studies-Depression Scale (CES-D; Radloff, 1977). Each item on the scale is rated on a 4-point Likert scale ranging from 0 (1 day or less, rarely or none of the time) to 3 points (5–7 days, most or all of the time) with a total score ranging from 0 to 60 points. A higher score indicates a higher level of depression. The internal consistency of this scale in this study was excellent (Cronbach’s α = .90).

#### Fear of COVID-19 Scale (FCV-19S)

Fear perceived by dual-income couples during COVID-19 was measured using the Korean version of the questionnaire (M. H. Seong et al., 2020) adapted from the Fear of COVID-19 Scale (FCV-19S; Ahorsu et al., 2022). This 5-item scale was developed to investigate COVID-19-related fear. Each item is rated on a 5-point Likert scale (1 = strongly disagree, 2 = disagree, 3 = neutral, 4 = agree, 5 = strongly agree). The total score ranges from 7 to 35, with a higher score indicating a higher level of fear of COVID-19. The internal consistency of this scale in this study was good (Cronbach’s α = .76).

#### Work–Family Behavioral Role Conflict Scale

Work–family conflict perceived by dual-income couples during COVID-19 was measured using the Korean version of the questionnaire (M. Seong et al., 2020) of the Work–Family Behavioral Role Conflict Scale (WFBRC-S3; Clark et al., 2019). Each item of this 25-item scale is rated on a 5-point Likert scale (1 = strongly disagree, 2 = disagree, 3 = neutral, 4 = agree, 5 = strongly agree), whereby a higher score indicates a higher level of conflict. The internal consistency of this scale in this study was excellent (Cronbach’s α = .93).

### Statistical Analyses

The data in this study were analyzed using the SPSS/WIN 24.0 statistical program. Participants’ general characteristics and levels of depression, fear, and work–family conflict were analyzed using descriptive statistics (frequency, percentage, mean, and standard deviation). The *t*-test and ANOVA were used to test the differences in behavioral and psychological symptoms according to general characteristics. In accordance with the assumption of regression analysis, the correlations between independent variables were analyzed using Pearson correlation coefficients, and the influence of each factor associated with work–family conflict was analyzed using hierarchical regression analysis. The reliability of the instruments was analyzed with Cronbach’s α, and the adequate level was set to .70 or higher with reference to Nunnally (1978).

## Results

### Participants’ General Characteristics

Participants’ general characteristics are outlined in Table 1. Among the respondents, 60.3% were aged 40 to 50 years; 49.1% were male and 50.9% were female; 61.2% were university graduates; 52.8% had no religion; 57.9% were office workers and 91.1% were permanent employees; 43% had a monthly household income of at least 7 million won; 63.6% worked 40 hr or less a week; 50.0% had one child, with the most frequent age range for the youngest child being 9 to 12 years (32.7%). In terms of the sex distribution of the children, boys outnumbered the girls (55.1% vs. 44.9%); 73.4% received support for childcare and 26.6% had no childcare support.

**Table 1.** Differences in Work-Family Conflict by General Characteristics of Participants (*N* = 214).

Variables	*n* (%)	*M* ± *SD*	*t* or *F*
Age (year)			−0.517
≤30–39	85 (39.7)	76.80 ± 16.24	
≥40–50	129 (60.3)	78.02 ± 17.41	
Sex			−0.592
Male	105 (49.1)	76.84 ± 17.86	
Female	109 (50.9)	78.21 ± 16.03	
Education (year)			0.747
≤High school	5 (2.3)	78.40 ± 11.93	
College	35 (16.4)	74.63 ± 18.07	
University	131 (61.2)	77.36 ± 16.80	
≥Graduate school	43 (20.1)	80.35 ± 16.90	
Religion			−2.302*
Yes	101 (47.2)	75.04 ± 16.80	
No	113 (52.8)	80.33 ± 16.70	
Occupation			0.256
Management	24 (11.2)	77.42 ± 15.79	
Professional	35 (16.4)	77.00 ± 17.38	
Service industry	25 (11.7)	79.32 ± 16.63	
Office job	124 (57.9)	77.64 ± 16.97	
Blue-collar job	6 (2.8)	71.67 ± 22.99	
Employment type			0.478
Permanent position	195 (91.1)	77.32 ± 16.90	
Temporary position	17 (7.9)	78.76 ± 18.22	
Other	2 (1.0)	88.50 ± 3.54	
Monthly household income (won)			0.891
<300	4 (1.9)	81.50 ± 7.85	
300–399	13 (6.1)	77.23 ± 17.09	
400–499	25 (11.7)	72.08 ± 19.78	
500–599	38 (17.8)	75.61 ± 19.32	
600–699	42 (19.5)	79.79 ± 15.04	
≥700	92 (43.0)	78.66 ± 16.09	
Working hours per week (hr)			−2.073*
≤40	136 (63.6)	75.74 ± 17.60	
>40	78 (36.4)	80.68 ± 15.27	
Number of children			0.103
1	107 (50.0)	78.01 ± 15.22	
2	90 (42.1)	76.86 ± 18.70	
3	13 (6.1)	77.62 ± 19.97	
4	4 (1.8)	80.00 ± 12.38	
Age of youngest child			1.67
≤2	31 (14.5)	78.16 ± 19.90	
03-May	52 (24.3)	77.33 ± 14.06	
06-Aug	61 (28.5)	80.98 ± 18.05	
09-Dec	70 (32.7)	74.41 ± 16.18	
Sex of youngest child			0.202
Male	118 (55.1)	77.07 ± 17.76	
Female	96 (44.9)	78.11 ± 15.91	
Support for childcare			0.295
Yes	157 (73.4)	78.11 ± 16.39	
No	57 (26.6)	77.33 ± 17.16	

*Note. M* = mean; *SD* = standard deviation.

* *p* < .05.

### Differences in Work–Family Conflict Based on Participants’ General Characteristics

Table 1 presents the differences in work–family conflict among the participating dual-income parents based on their general characteristics. The variables that showed significant differences in work–family conflict among the participants were religion and weekly working hours. The level of work–family conflict was significantly higher in dual-income parents without religion than in those with religion (*t* = −2.302, *p* < .05). Those working more than 40 hr per week showed a higher level of work–family conflict than those working 40 hr or less (*t* = −2.073, *p* < .05).

### Correlations Between the Participants’ Depression, Fear, and Work–Family Conflict During the COVID-19 Pandemic

Table 2 presents the correlations between participants’ depression, fear, and work–family conflict during the COVID-19 pandemic. Depression was positively correlated with fear (*r* = .44, *p* < .01) and work–family conflict (*r* = .54, *p* < .01). Fear was positively correlated with work–family conflict (*r* = .43, *p* < .01).

**Table 2.** Correlation Between Depression, Fear and Work-Family Conflict (*N* = 214).

Variables	1	2	3
1. Depression	1		
2. Fear	0.44**	1	
3. Work–Family conflict	0.54**	0.43**	1
Mean	20.03	17.98	77.54
Standard deviation	11.07	6.15	16.92
Minimum	2.00	7.00	31.00
Maximum	54.00	32.00	119.00
Range	0–60	7–35	25–125
Skewness	0.52	0.28	–0.40
Kurtosis	–0.57	–0.70	0.12

** *p* < .01.

### Predictors of the Participants’ Work–Family Conflict During the COVID-19 Pandemic

To determine the effects of the major factors that influence work–family conflict in dual-income parents during the COVID-19 pandemic, we selected religion, working hours per week, depression, and fear as independent variables, which led to statistically significant differences in or were significantly correlated with the level of work–family conflict. The normality analysis of the selected variables led to the following findings: skewness and kurtosis met the assumption of normal distribution with absolute values meeting the criteria of less than 3 and 10, respectively (Table 2). The correlation between the independent variables was <.80. With the variance inflation factor (VIF) ranging from 1.00 to 1.25, and thus not exceeding 10, there was no problem of multicollinearity. With the major variables showing a linear relationship and the standardized residual and Cook’s distance not exceeding the absolute value of 3 and 1.0, respectively, there were no outliers. Likewise, with a Durbin-Watson value of 1.86, which is close to the standard value of ±2, there was no autocorrelation between variables, which indicates no infringement of the independence of the error term. All the basic assumptions of the regression model for performing the regression analysis were satisfied.

The first stage of the hierarchical regression analysis included two general characteristic variables: religion and working hours per week, of which the former was significantly correlated with work–family conflict (β = .15, *p* < .05). This model was statistically significant (*F* = 4.59, *p* < .05), and the explanatory power was 3%. In the second stage, where depression was included, working hours per week (β = .13, *p* < .05) and depression (β = .53, *p* < .001) were significantly correlated with work–family conflict. This model was statistically significant (*F* = 32.81, *p* < .001), and the explanatory power was 31%. In the third stage, where fear was included, working hours per week (β = .12, *p* < .05), depression (β = .43, *p* < .001), and fear (β = .23, *p* < .001) were significantly correlated with work–family conflict. This model was statistically significant (*F* = 29.80, *p* < .001), and the explanatory power was 35% (Table 3).

**Table 3.** Predictors of Work-Conflict of Dual-Income Parents During the COVID-19 Pandemic (*N* = 214).

Variables	Model 1		Model 2		Model 3	
	B	β	B	β	B	β
Religion^a^	5.01	.15*	3.24	.10	3.29	.10
Working hours per week (hr)^b^	4.62	.13	4.66	.13*	4.23	.12*
Depression			0.81	.53***	0.65	.43***
Fear					0.64	.23***
*R* ^2^	.04		.31		.36	
Adj *R*^2^	.03		.31		.35	
*F* (*p*)	4.59*		32.81***		29.80***	

*Note*. B = unstandardized coefficients; β = standardized coefficients.

a Dummy variables (Yes = 1, No = 0).

b Dummy variables (working hours >40 hr = 1, ≤40 hr = 0).

* *p* < .05, ***p < .001.

## Discussion

This study determined the effects of depression and fear in dual-income parents on work–family conflict during the COVID-19 pandemic. Two general characteristic variables (religion and working hours per week), which are significantly associated with work–family conflict, and the variables depression and fear were subjected to statistical testing.

Participants’ mean depression score was 20.03. Considering that a CES-D scale score of 16 or higher is classified as mild or possible depression, the participants were confirmed to have a clinical level of depression requiring intervention. In a study conducted before the outbreak of COVID-19 (Yeom & Yang, 2019), the mean CES-D score of 210 mothers of preschool children was 13.71, much lower than that of this study. By way of comparison, gender-dependent differences in depression scores may be considered given that the participants of the previous study were exclusively women. However, not only is there a higher prevalence of depression in women than men (Parker & Brotchie, 2010), but a study on gender differences in depression conducted with about 6,700 Koreans also verified that women are also more vulnerable to it (Gitto et al., 2015). From these results, it can be inferred that the level of depression perceived by the participants in this study is higher than that in the previous study that only sampled women. The difference may be attributed to the fact that this study was conducted at a time when the COVID-19 pandemic had become unrelenting and aggravating. Specifically, Shevlin et al. (2020) reported that the level of depression reported in studies conducted during the pandemic increased compared to that in studies conducted before the outbreak, and that the presence of children at home is a predictor of depression. Fukase et al. (2021) also reported that the prevalence of depression during the pandemic was two to nine times higher than that in the pre-COVID-19 era. Moreover, depression is expected to intensify in dual-income parents who must grapple with the double burden of maintaining work–family balance if they respond poorly to the childcare gap. This highlights the need to provide dual-income couples with systematic programs for depression management and, at the same time, social support to narrow the childcare gap especially when infectious diseases, such as COVID-19, become pandemics and unrelenting threats.

Participants’ mean fear score was 17.98. There is little to no research dedicated to measuring fear in dual-income parents during the pandemic. For this reason, the fear score of 2.99 measured in this study was much higher than that measured at the time of validity and reliability testing of the fear scale used in this study. It has also been reported that the COVID-19 pandemic evokes negative emotions, such as worry and fear, in most parents (Ares et al., 2021). Therefore, even under the same circumstances, the level of fear may be higher in the current study’s participants compared with those of the previous one, which covered age groups from teens to older adults (60+) and included unmarried participants (≥ 40%). This may be partially supported by the research finding that fear of COVID-19 is significantly associated with mental health outcomes in socially vulnerable populations, such as families with children (Fitzpatrick et al., 2020). Along with the recent changes in our society, the number of dual-income families with children is increasing. With the end of the COVID-19 pandemic nowhere in sight, it is understandable that the uncertainty surrounding the pandemic creates greater fear in dual-income couples who have children under their care and protection. Nevertheless, in view of the lack of relevant research worldwide, the results of this study will have to be further scrutinized in future studies.

The hierarchical regression analysis of this study identified the predictors of work–family conflict, particularly depression, fear, and working hours per week in decreasing order of importance, in dual-income parents during the COVID-19 pandemic. In this study, participants with a higher level of depression showed a higher level of work–family conflict. A comparable study by Hong and Lee (2020) with different participants (working mothers with early school-age children in Korea) found that depression determines the type of change in work–family conflict. Likewise, Guille et al. (2017) reported an association between work–family conflict and an increase in depressive symptoms, partially supporting the results of this study. While having sampled different participants, these studies have identified depression as a predictor of work–family conflict; it is therefore incumbent on our society to detect depression at an early stage and actively manage it (Dinakaran et al., 2020). Such measures could be an outlet for dual-income parents to regain psychological stability, improve psychological well-being, and resolve work–family conflict. While the main focus of most previous studies has been the effect of work–family conflict on depression, this study reversed that focus, examining instead the effect of depression on work–family conflict. Nohe et al. (2015) examined whether work–family conflict predicts tension or whether tension predicts work–family conflict, and reported that they are intercorrelated. They suggested that it is essential to investigate which viewpoint is empirically justified in terms of the challenges presented by the traditional monodirectional perspective thus far. In this context, repeat studies are needed to further justify the findings of this study in its attempt to test the research question in a direction different from that of previous studies.

In this study, participants with a higher level of fear during the COVID-19 pandemic showed a significantly higher level of work–family conflict. The literature review of previous studies with dual-income parents did not yield conclusive results to fully support the findings of this study. However, fear of COVID-19 was found to increase family harm, that is, conflicts between family members in the family domain (Sit et al., 2021), which has been found to decrease job satisfaction and increase turnover intention in the work domain (Labrague & de Los Santos, 2021). These findings provide partial evidence for the results of this study. At present, the COVID-19 pandemic has made it difficult to find solace and optimism, and has disrupted our personal and professional lives (Reio, 2020). During such a time, overcoming our fears and maintaining work–life balance becomes even more important. Based on the results of this study, it is necessary to prepare various intervention programs in this respect, which can help dual-income couples overcome fear and resolve work–family conflicts. It is also necessary to reinforce government-led disaster psychological support, such as counseling, education, and mental health management services, to help dual-income parents overcome their fear of the COVID-19 pandemic.

The number of working hours per week in this study was statistically positively correlated with the level of work–family conflict. Specifically, those who worked more than 40 hr per week showed a higher level of work-family conflict compared with those who worked 40 hr per week or less (legal working hours in Korea). Schooreel and Verbruggen (2016) reported that work–family conflict in dual-income couples decreased when weekly working hours were reduced, which is in line with the findings of this study. Another study conducted with working mothers also reported that an increase in working hours was associated with more work–family conflict (Huang et al., 2020), which also supports the findings of this study. This highlights the importance of considering weekly working hours as an approach to preventing work–family conflict in dual-income parents. In particular, the issue of working hours is assumed to be directly related to work–family conflict for dual-income parents. Therefore, it is necessary to quantitatively expand workplace support, such as flexible working arrangements, and revamp the policy system that can facilitate their treatment. Moreover, we propose a government-level intervention to resolve the issue of working hours of double-income parents since the number of work hours is also related to personal life satisfaction (Ryu, 2015).

## Conclusion

The persistent spread of COVID-19 has exposed dual-income parents to emotional limitations in their efforts to maintain their identity and manage multiple roles for work–family balance in an unprecedented way. In this context, this study sought to uncover predictors of work–family conflict with a focus on the emotional limitations dual-income parents experience during the COVID-19 pandemic. From a psychological perspective, the findings of this study are expected to serve as a starting point for resolving the work–family conflict of dual-income parents across the globe who are coping with negative emotions amid the pandemic.

Caution is warranted in interpreting the results of this study in relation to the following limitations: First, they cannot be generalized to all dual-income parents since the study was only conducted on dual-income parents with preschool and primary school children. Second, gender differences were not considered in this study even if they may exist in work–family conflict (Allen & Finkelstein, 2014), because it was conducted on dual-income couples. Third, the main focus was the psychological aspects of dual-income parents, leaving various factors, such as physical and environmental factors, unexplored. Fourth, caution should be taken when extending the study’s results to the pre-COVID-19 period since this study was specifically conducted under COVID-19 circumstances. Despite these limitations, the significance of the study lies in the following facts: it reflects the unusual circumstances of the COVID-19 pandemic and elaborates on depression and fear as a research topic. The study seeks to identify the psychological factors affecting work–family conflict by reverse-testing the conventional research direction. In addition, it provides a theoretical basis for psychological intervention to promote work-family conflict management and an empirical basis for setting up a comprehensive support plan for dual-income families with preschool and primary school children.

This study found that both depression and fear are perceived by dual-income parents as factors contributing to increasing work–family conflict during the pandemic. An increase in working hours per week was also identified as a risk factor for work–family conflict in dual-income parents. Above all, depression was identified as the strongest factor associated with the work–family conflict of dual-income parents during the pandemic. This highlights the need to carry out active depression management and adopt a systematic approach to prevent the incidence of depression.

Based on the above findings, we propose the following research directions in exploring work–family conflict. First, the age range of dual-income family children must be diversified and its relevance to work–family conflict tested. Second, repeat studies considering the sex of dual-income parents should be conducted and sex differences in work–family conflict investigated. Third, follow-up research must be undertaken to identify the various factors affecting the work–family conflict of dual-income parents and to test their relevance. Finally, various programs for mental health management and national policy-making support as multidimensional and systematic strategies must be developed for effective intervention with dual-income parents exposed to depression and fear.
